# Potential Antimicrobial Properties of Coffee Beans and Coffee By-Products Against Drug-Resistant *Vibrio cholerae*

**DOI:** 10.3389/fnut.2022.865684

**Published:** 2022-04-25

**Authors:** Anchalee Rawangkan, Achiraya Siriphap, Atchariya Yosboonruang, Anong Kiddee, Grissana Pook-In, Surasak Saokaew, Orasa Sutheinkul, Acharaporn Duangjai

**Affiliations:** ^1^School of Medical Sciences, University of Phayao, Phayao, Thailand; ^2^Unit of Excellence in Research and Product Development of Coffee, Division of Physiology, School of Medical Sciences, University of Phayao, Phayao, Thailand; ^3^Division of Social and Administrative Pharmacy, Department of Pharmaceutical Care, School of Pharmaceutical Sciences, University of Phayao, Phayao, Thailand; ^4^Center of Health Outcomes Research and Therapeutic Safety (Cohorts), School of Pharmaceutical Sciences, University of Phayao, Phayao, Thailand; ^5^Unit of Excellence on Clinical Outcomes Research and IntegratioN (UNICORN), School of Pharmaceutical Sciences, University of Phayao, Phayao, Thailand; ^6^Faculty of Public Health, Mahidol University, Bangkok, Thailand

**Keywords:** antimicrobial activity, coffee by-products, coffee extract, drug-resistant, *Vibrio cholerae*

## Abstract

*Vibrio cholerae* is the causative organism of the cholera epidemic, and it remains a serious global health problem, particularly the multidrug-resistant strain, despite the development of several generic drugs and vaccines over time. Natural products have long been exploited for the treatment of various diseases, and this study aimed to evaluate the *in vitro* antibacterial activity of coffee beans and coffee by-products against *V. cholerae* antimicrobial resistant strains. A total of 9 aqueous extracts were investigated, including light coffee (LC), medium coffee (MC), dark coffee (DC), dried green coffee (DGC), dried red coffee (DRC), fresh red coffee (FRC), Arabica leaf (AL), Robusta leaf (RL), and coffee pulp (CP). The influential coffee phytochemicals, i.e., chlorogenic acid (CGA), caffeic acid (CA), and caffeine, were determined using HPLC. The antibacterial properties were tested by agar well-diffusion techniques, and the minimum inhibitory concentration (MIC) and minimum bactericidal concentration (MBC) were further determined against 20 *V. cholerae* isolates. The results revealed that all tested strains were sensitive to coffee extracts, with MIC and MBC values in the range of 3.125–25.0 mg/mL and 12.5–50.0 mg/mL, respectively. With a MIC of 6.25 mg/mL, DGC, DRC, and CP appeared to be the most effective compounds against 65, 60, and 55% of clinical strains, respectively. The checkerboard assay revealed that the combination of coffee extract and tetracycline was greater than either treatment alone, with the fractional inhibitory concentration index (FICI) ranging from 0.005 to 0.258. It is important to note that CP had the lowest FICI (0.005) when combined with tetracycline at 60 ng/mL, which is the most effective dose against *V. cholerae* six-drug resistance strains (azithromycin, colistin, nalidixic acid, sulfamethoxazole, tetracycline, and trimethoprim), with a MIC of 47.5 μg/mL (MIC alone = 12.5 mg/mL). Time killing kinetics analysis suggested that CA might be the most effective treatment for drug-resistant *V. cholerae* as it reduced bacterial growth by 3 log_10_ CFU/mL at a concentration of 8 mg/mL within 1 h, via disrupting membrane permeability, as confirmed by scanning electron microscopy (SEM). This is the first report showing that coffee beans and coffee by-product extracts are an alternative for multidrug-resistant *V. cholerae* treatment.

## Introduction

Cholera is an acute diarrheal infection caused by the consumption of contaminated food or water containing the gram-negative bacteria *Vibrio cholerae*, especially the serogroups O1 and O139, which are capable of causing cholera outbreaks that can kill within hours if left untreated. Seven cholera pandemics have already been reported throughout the world (1). According to the most recent global burden estimate, there are approximately 1.3–4.0 million cholera cases per year, with 21,000–143,000 deaths worldwide (2). Despite the availability of a vaccine, 923,037 cases were reported from 31 countries in 2019, with 1,911 deaths (a mortality rate of 0.2%) (3). According to World Health Organization (WHO) reports, a global cholera control strategy called “Ending Cholera: A Global Roadmap to 2030” was created with the goal of reducing the mortality rate by 90% (4).

Oral rehydration therapy, supplemented with antibiotics such as tetracycline, fluoroquinolones, and azithromycin, is the primary treatment for *V. cholerae* (5). Due to its extraordinary genomic plasticity, treatment failures have become more common in recent years, with the recurrence of antimicrobial resistant *V. cholerae* (6–11). The rise of drug-resistant *V. cholerae* is a major public health concern because the illnesses that occur are often more severe and difficult to treat. Infections with drug-resistant *V. cholerae* lead to greater mortality rates, longer hospital stays, more secondary infections, and higher medical expenses (12). In Thailand, 61.5% (48 of 78 isolates) of *V. cholerae* isolates between 1991 and 2013 were reported to be antimicrobial resistant strains, with 56.3% of them being multidrug-resistant (MDR) and conferring resistance to three or more antimicrobial classes (13). It is important to note that the development of antibiotic resistance outpaces the development of new drugs, resulting in a global problem with long-term negative consequences. Therefore, the development of new anti-*Vibrio* compounds, particularly those derived from plants, has become critical.

Natural compounds against *V. cholerae* have been shown to inhibit bacterial growth or the secreted cholera toxin, including catechins from green tea (*Camellia sinensis*) (14), procyanidins from *Guazuma* (*Guazuma ulmifolia*) (15), gallate analogs from Daio (*Rhei rhizoma*) (16), apelphenon from apple (*Malus* spp.) (17), procyanidins from hop (*Humulus lupulus*) (18), oil (diallyl sulfides) from elephant garlic (*Allium ampeloprasum*) (19), and capsaicin from red chili (*Capsicum annum*) (20, 21). Piperidine, chlorogenic acid (CGA), and eugenyl acetate derived from *Piper betel* have also been shown to be equally effective against MDR strains of *V. cholerae* (22–24). Carvacrol, a major essential oil fraction of Oregano (*Origanum vulgare*), inhibited the virulence of *V. cholerae* by inhibiting mucin penetration, adhesion, and the expression of virulence-associated genes (*tcpA, ctxB, hlyA*, and *toxT*), resulting in reduced fluid accumulation (25). On the other hand, cranberry (*Vaccinium macrocarpon*) extract inhibited *V. cholerae* biofilm formation, possibly by modulating the cyclic dimeric guanosine monophosphate (c-di-GMP) level (26). Furthermore, methanolic extracts of basil (*Ocimum basilicum* L.), nopal cactus (*Opuntia ficus-indica* var. Villanueva L.), sweet acacia (*Acacia farnesiana* L.), and white sagebrush (*Artemisia ludoviciana* Nutt.) were found to be the most active against *V. cholerae* via cell membrane disruption (27). However, there has been no mention of coffee extract.

Coffee (*Coffea* L.) is one of the world's most valuable primary products (28). *C. arabica* L. cv. Caturra (Arabica) is the most popular and preferred coffee cultivar worldwide. Coffee processing generates a large amount of solid by-products during coffee cultivation and preparation, such as spent coffee grounds, the by-products of coffee fruit and bean processing (coffee husks, peel, pulp), and so on (29). Recently, we revealed that coffee beans or coffee by-product extract, which are high in phenolic compounds and antioxidant activity, seem to have a wide-range of health benefits, including anti-hyperglycaemic and anti-hyperlipidaemic activities (30), anti-adipogenic and lipolytic properties (31), anti-diabetic, cholesterol-lowering, and anti-hepatic steatosis activity (32–34), anti-hepatic steatosis activity (35, 36), as well as antibacterial activity against both gram-positive and gram-negative bacteria (37). Therefore, the use of coffee extracts with medicinal properties could be an alternative treatment for various diseases.

Coffee beans contain a variety of compounds with powerful bioactive activities, i.e., caffeine, CGA, diterpenes, and trigonelline (38). Several studies have shown that coffee extracts have strong antibacterial activity (39–42). Flavonoids, CGA, caffeic acid (CA), trigonelline, caffeine, and protocatechuic acid play a key role as potential natural antimicrobial agents against enteric bacteria (40, 43, 44), but there is no relevant data on *V. cholerae*. Nevertheless, the efficacy varies, depending on the species, degree of roasting, brewing procedure, and decaffeination (45). Coffee varieties from various origins differ significantly in terms of their constituents, and multiple agricultural geography conditions of the coffee plant, such as the soil type, altitude, and harvest season, as well as the pre- and post-harvest management practices, influence coffee bean bioactivity (46, 47).

For the first time, we shed light on the potential antimicrobial properties of coffee beans and coffee by-products against MDR *V. cholerae* in health improvement treatments. With this goal, this work seeks (1) to examine the antimicrobial activity of coffee beans, classified by temperature and roasting time, as well as coffee by-product extracts, such as coffee fruits and leaf extracts, and coffee pulp (CP) extract, (2) to investigate the synergistic effects of the crude extract compounds of coffee with the antibiotic tetracycline, and (3) to assess the pharmacological mode of action of coffee bioactive molecules with respect to potential disruption in the membrane of microorganisms and their effect on bacterial morphology, which may be helpful to bring about new opportunities in complementary and alternative medicine.

## Materials and Methods

### Preparation and Phytochemical Characterization of Coffee Beans and Coffee By-Products

#### Plant Materials and Extract Preparation

The Chao-Thai-Pukao Factory (Chiang Mai, Thailand) provided coffee beans and coffee by-products. As indicated in the previous report, NU003806 was the coffee tree's voucher number (30).

#### Roasted Coffee Extracts: Light Coffee, Medium Coffee, and Dark Coffee

The roasted coffee extracts were prepared from green coffee beans (*Coffea arabica* L.), with the degree of roasting performed in accordance with previous studies (30, 48). Light coffee (LC), medium coffee (MC), and dark coffee (DC) are classified by the roasting temperature and roasting time (176.7–232.2°C and 10–20 min). The roasted coffee was extracted with water (1:5; w/v) using an ultrasonic bath at 35 kHz at 40°C for 5 min. The filtered samples were dried using a freeze dryer (CoolSafe 110-4 Pro, LaboGeneTM, Allerød, Denmark), and the LC, MC, and DC extracts were then stored at −20°C for further study.

#### Coffee Fruit Extracts: Dried Green Coffee, Dried Red Coffee, and Fresh Red Coffee

Coffee fruit extracts were prepared according to previous studies (31, 32). Briefly, fresh and dried coffee fruits were extracted with boiling distilled water for 30 min (1:10; w/v). The aqueous solution was dried by a freeze dryer (CoolSafe 110-4 Pro, LaboGeneTM, Allerød, Denmark), and the powder of dried green coffee (DGC), dried red coffee (DRC), and fresh red coffee (FRC) were stored at −20°C until use.

#### Coffee Leaf Extracts: Arabica Leaf and Robusta Leaf

*Coffea arabica* L. cv. Caturra (Arabica) and *C. canephora* var. robusta (Robusta) leaves were extracted with boiling water (1:5; w/v) for 10 min. This step was repeated three times, and then the filtered solutions were freeze dried. The powder was stored at −20°C until further examination.

#### Coffee Pulp Extract

The coffee pulp (CP) was extracted according to a previous study (37). Briefly, dried pulps were extracted with boiling water (1:5; w/v) for 10 min. This step was repeated twice before the solutions were freeze dried. The pulp powder was stored at −20°C for later use.

#### Determination of Coffee Phytochemical Content by Chromatographic Analysis

Six coffee extracts were subjected to high-performance liquid chromatography (HPLC) to determine the levels of CGA, CA, and caffeine, according to previous studies (31, 48). In brief, the HPLC separation of the LC, MC, DC, CP, AL, and RL extracts was performed on a C18 column (4.6 × 150 mm, 5 μm) using mobile phase A (15% methanol) and mobile phase B (85% methanol:distilled water [30:70], 2% acetic acid; pH 3.4). The flow rate was set at 0.5 mL/min for 30 min, with detection at 280 and 320 nm for CA, caffeine, and CGA. The peaks were identified by the reference standards. DGC, DRC, and FRC extracts were run with 0.1% formic acid in water (mobile phase A) and 0.1% formic acid in acetonitrile (mobile phase B) using HPLC and coupled to LC-ESI-Q-TOF-MS according to previously reported (31).

### Bacterial Strains and Growth Conditions

The clinical strains of 20 representative isolates of *V. cholerae* serogroups O1 and O139 were obtained from previous studies (13), in which they were isolated from feces and rectal swabs of patients in Thailand between 1994 and 2004. The 7th pandemic *V. cholerae* N16961 strain from Bangladesh in 1975 was used as a standard reference strain. The antibiotic resistance pattern of all strains has previously been characterized and can be found in Supplementary Table 1.

To perform the preliminary antimicrobial screening of the effect of each crude extract on *V. cholerae* growth inhibition, the N16961 strain was grown overnight in Mueller Hinton Broth (MHB) containing 1% NaCl at 37°C. The 0.5 McFarland turbidity standard cultures (1–1.5 × 10^8^ colony-forming units; CFU) were spread onto Mueller Hinton Agar (MHA) plates using sterile cotton swabs, according to the Clinical and Laboratory Standards Institute (CLSI) guidelines (49). The extracted compounds were diluted in MHB. Then, 50 μL of filtered extracts were allowed to diffuse into a 6-mm cork borer well in MHA containing 1% NaCl medium at 500 mg/mL. The plates were kept at room temperature for 30 min to allow diffusion of the test solution into the surrounding media. The plates were then incubated at 37°C for 18 h. Each plate was examined for the inhibition zone. Tetracycline, the first line treatment for cholera disease, was used as a positive control at a concentration of 30 μg/mL, and media solution was used as a negative control (50).

### Determination of the Minimum Inhibitory Concentration and the Minimum Bactericidal Concentration

The minimum inhibitory concentration (MIC) values were determined using a 96-well microtiter plate and the CLSI protocol (51, 52). Freshly prepared stock solutions of the extracts or their phytochemical compounds were serially diluted twice using MHB with 1% NaCl. All wells were inoculated with *V. cholerae* at a final volume of 100 μL of bacterial inoculum (5 × 10^5^ CFU/mL). After incubation for 24 h at 37°C, 1 mg/mL resazurin was added to all wells (10 μL per well), and the plates were further incubated for 4 h to observe the color change. On completion of the incubation, columns with no color change (blue resazurin color remained unchanged) were scored as being above the MIC value (53).

The MBC was determined using the MHA plates with 1% NaCl by dropping 10 μL of test solution directly into the content of the wells that had concentrations higher than the MIC value, and then incubating at 37°C for 24 h. The MBC value was determined when there was no colony growth from the contents of the 10 μL directly-plated wells. In addition, the contents of the wells showing indications of growth inhibition were serially diluted to quantify the end-point killing of the bacteria, as detailed in the results section.

### Antimicrobial Synergy Testing

The checkerboard assay was used to determine the potential synergistic activity of the extracts and tetracycline on *V. cholerae* N16961 and P48 *V. cholerae* El Tor Ogawa strains, which are a reference and tetracycline resistance strain, respectively (54). The extract compounds were serially diluted to 1/128 MIC, while the drug was serially diluted to 1/516 MIC. Compounds and antibiotics were prepared in 96-well microtiter plates using 2-fold serial dilutions based on the MIC of each substance. A final bacterial suspension at 5 × 10^5^ CFU/mL was added to each well. After incubation for 24 h at 37°C, the wells were visually inspected, and the synergistic MIC (compound in combination with antibiotic) was determined as the first well with no visible turbidity. The observed MIC values were used to calculate the fractional inhibitory concentration index (FICI), which allows evaluation of the combined effects of an antibiotic and a compound according to the following formula: FICI = FIC (a) + FIC (b), where FIC (a) = MIC of extract in the combination/MIC of extract alone, and FIC (b) = MIC of tetracycline in the combination/MIC of tetracycline alone. These values were interpreted as follows: for FICI ≤0.5: a synergistic effect; for FICI >0.5 and ≤4: an additive effect; and for FICI >4: an antagonistic effect (55, 56).

### Time-Kill Kinetics Assay

The killing kinetics of the potent coffee phytochemical compounds, including CGA, CA, and caffeine, at 1x, 2x, 4x, and 8x MIC values were determined using the method described previously (57–59), with slight modifications. Different concentrations of compounds were added to reach the final volume of 100 μL with 1 × 10^5^ CFU/mL of *V. cholerae* N16961 reference stain grown in MHB containing 1% NaCl and kept at 37°C. Bacterial growth was monitored over a time-course of 24 h (0, 1, 2, 4, 8, 16, 24 h). A sample without the compound served as a growth control. To evaluate the survival of the pandemic strains during the observation period, aliquots of serial dilutions of the bacterial suspensions were determined by a spread plate technique on MHA with 1% NaCl, and the plates were incubated at 37°C for 24 h to evaluate the viable bacterial colony counts. Data was analyzed as killing curves by plotting the log_10_ CFU/mL vs. time (h), and the change in bacterial concentration was determined. The viable bacterial cell count for the time-kill end point determination, i.e., bactericidal activity, was defined as a reduction of ≥3 log_10_ CFU/mL relative to the initial inoculum, whereas bacteriostatic activity corresponded to a <3 log_10_ CFU/mL decrease relative to the initial inoculum (60).

### Outer Membrane Permeabilization Analysis

#### Determination of Nucleotide and Protein Leakage

The leakage of cytoplasmic elements from the cell was used to evaluate the integrity of the cell membrane using the method described by Lou et al. (61), with some modifications. In brief, the *V. cholerae* N16961 cells were cultured overnight at 37°C, and the cells were washed and resuspended at a concentration of 1 × 10^7^ CFU/mL in phosphate buffer saline (PBS), pH 7.2. Then, 1 mL of these suspensions was incubated with CGA, CA, and caffeine at concentrations of 1, 2, 4, and 8x MIC at 37°C for 1 h. After centrifugation, the supernatant samples were immediately filtered through a 0.2 μm organic membrane, and the optical density measured at 260 nm using a NANO-400A Micro Spectrophotometer, to determine the amounts of DNA released from the cytoplasm. The cell integrity was further examined by determining the release of proteins into the supernatant. The Bradford dye-binding reagent of the Bio-Rad DC Protein Assay kit (Bio-Rad Laboratories, Inc., USA) was used to determine the amount of protein by measuring the optical density of the resulting solution at 750 nm within 5 min. The protein quantity of each sample was determined from the equation of the best-fit linear regression obtained from the Bovine Serum Albumin (BSA) standard curve. Triton X-100 (0.1%; v/v) was used as a positive control, while PBS inoculated with the same inoculum was used as a negative control.

#### Determination of Outer Membrane Disruption

The effect of the potent coffee phytochemical compounds on the bacterial outer membrane permeability was determined using an N-Phenyl-1-naphthylamine (NPN) uptake assay (62, 63). Briefly, *V. cholerae* N16961 cells were treated with 0, 1, 2, 4, and 8 MIC at a final volume of 1 mL and incubated for 1 h at 37°C. The cell suspensions were then washed and resuspended in 1 mL of 0.5% NaCl. NPN solution (TCI, Japan) in ethanol (100 mM) was added to 200 μL of cells to give a final concentration of 0.75 mM. The background fluorescence was recorded for subtraction, using the Cytation 5 Cell Imaging Multi-Mode Reader with an excitation wavelength of 350 nm and an emission wavelength of 420 nm at room temperature. As the outer membrane permeability increased due to the addition of the coffee phytochemical compound, NPN incorporated into the membrane resulted in an increase in fluorescence. Triton X-100 (0.1%; v/v) was used as a positive control for the 100% maximum dye leakage release. Values were converted to % NPN uptake using the following equation: % NPN uptake = (Fobs-F0)/(F100-F0)x100, where Fobs is the observed fluorescence at a given compound concentration, F0 is the initial fluorescence of NPN with the cells in the absence of compound, and F100 is the fluorescence of NPN with the cells upon addition of TritonX-100 (64).

#### Determination of Cell Membrane Potential

To measure the changes in membrane polarity caused by the coffee, bioactive compounds were adapted through the incorporation of Rhodamine 123 (Rh123) (Sigma-Aldrich, USA) (65–67). *V. cholerae* N16961 cells were treated with 0, 1, 2, 4, and 8 MIC at a final volume of 1 mL and incubated for 1 h at 37°C. The cell suspension was mixed with a freshly-prepared Rh123 solution (final Rh123 concentration, 5 μg/mL), kept at 37°C for 10 min, and centrifuged at 1,500 rpm for 10 min. The cell pellets were then diluted in 0.5% NaCl, and the fluorescence signal measured at the excitation and emission wavelengths of 480 and 530 nm, respectively. The fluorescence intensity were calculated using the equation: Relative fluorescence intensity = F1/F0 × 100%, where F0 is the fluorescence intensity of untreated cells, and F1 is the fluorescence intensity of CA-treated cells.

### Analysis of Scanning Electron Microscopy

*Vibrio cholerae* N16961 was treated with CA at a concentration of 8x MIC for 2 h at 37°C. The appropriate treatment was harvested by centrifugation at 5,000 rpm for 5 min, washed with PBS, dropped onto a filter membrane of 0.2 μm, and air dried. The samples were fixed using 2.5% (v/v) glutaraldehyde in PBS at 4°C overnight. Thereafter, the bacteria were washed with 0.1M PO_4_ buffer and re-fixed with 1% OsO_4_ for 1 h. After dehydration with a graded ethanol series (50, 70, 90, and 100%) for 10 min each, the bacterial samples were transferred to absolute ethanol for 20 min. After drying by critical-point drying (CPD), the bacterial sample was mounted and coated with gold, before examination by scanning electron microscopy (SEM) (JSM 5910 LV, Oxford Instrument) (62).

### Statistical Analysis

Values are presented as the mean ± standard deviation (SD) of three independent experiments. The significance of differences between the average values of different experimental treatments and controls was assessed by ANOVA, considering that statistical significance was set at a *p* < 0.05. When ANOVA revealed significant differences among treatments, *post-hoc* tests were carried out with Dunnett's Multiple Comparison Test from GraphPad Prism 5.01 (GraphPad Software, Inc., La Jolla, CA, USA).

## Results

### Characterization of Coffee Beans and Coffee By-Products

As indicated in Table 1, coffee beans and coffee by-products including the roasted beans, fruits, leaves, and pulp extracts, represented bioactive substances with CGA, CA, and caffeine. Calibration curves were linear over a large concentration range of 3.125–400 μg/mL for caffeine and CGA, and 3.125–200 μg/mL for CA, and exhibited good linear regressions (*r*^2^ = 0.9997 for caffeine, *r*^2^ = 0.9989 for CGA, *r*^2^ = 0.9986 for CA), data not shown. CGA was found to be more abundant in CP (13.45 mg/g extract), DGC (12.56 mg/g extract), and RL (12.04 mg/g extract) than in the other extracts. Additionally, roasted coffee had higher levels of CA than coffee leaves, pulp, and fruits. CA (2.66 mg/g extract) was detected in higher concentrations in LC than in the other extracts. Interestingly, all extracts, especially MC, had a high caffeine content (13.08–26.80 mg/g extract).

**Table 1:** The phytochemical profile of the extracts of coffee beans and coffee by-products.

**Samples**	**CGA** **(mg/g extract)**	**CA** **(mg/g extract)**	**Caffeine** **(mg/g extract)**
**Roasted coffee extracts**			
LC	11.21	2.66	23.39
MC	5.53	1.20	26.80
DC	2.69	1.01	22.77
**Coffee fruit extracts** ^*^			
DGC	12.56^*^	0.25^*^	ND
DRC	7.21^*^	0.21^*^	ND
FRC	6.97^*^	0.08^*^	ND
**Coffee leaf extracts**			
AL	1.99	0.80	17.72
RL	12.04	1.85	13.08
**CP extract**	13.45	1.10	16.88

*LC, light coffee; MC, medium coffee; DC, dark coffee; DGC, dried green coffee; DRC, dried red coffee; FRC, fresh red coffee; AL, Arabica leaf; RL, Robusta leaf; CP, coffee pulp; CGA, chlorogenic acid; CA, caffeic acid; ND, not determined*.

* *Our previous report (31)*.

As a result, the extract of coffee beans and coffee by-products containing coffee phytochemicals were used to determine the biological activity of the antibacterial analysis in future studies.

### The Extracts of Coffee Beans and Coffee By-Products Inhibit Drug Resistant *V. cholerae* Strains

The 7th pandemic N16961 reference strain was used to screen the growth inhibition effect of each extract on *V. cholerae*. Table 2 shows the diameter of the inhibition zones in treatments with 500 mg/mL of the extract. The inhibition zones range from 10.67 ± 3.79 to 16.67 ± 1.15 mm in the agar well-diffusion assay. Treatment with AL extract inhibited bacterial growth the most effectively, followed by CP and MC, respectively. The inhibition zones of AL, CP, and MC were 16.67 ± 1.15, 13.33 ± 1.53, and 13.00 ± 1.00 mm, respectively, which is approximately half that of tetracycline (26.00 ± 1.73 mm). Therefore, the extracts may have the potential to act as natural antibiotics.

**Table 2:** Diameters of inhibition zones obtained with coffee beans and coffee by-products at 500 mg/mL on the 7th pandemic *V. cholerae* O1 El Tor N16961 strain.

**Samples**	**Diameters of inhibition zones (mm)**
**Roasted coffee extracts**	
LC	12.33 ± 0.58^**^
MC	13.00 ± 1.00^**^
DC	11.67 ± 1.15^*^
**Coffee fruits extracts**	
DGC	11.33 ± 4.04^*^
DRC	11.00 ± 2.65
FRC	10.67 ± 3.79
**Coffee leaf extracts**	
AL	16.67 ± 1.15^***^
RL	12.00 ± 1.00^*^
**CP extract**	13.33 ± 1.53^**^
**Controls**	
Tetracycline	26.00 ± 1.73^***^
MHB	6.00 ± 0

*LC, light coffee; MC, medium coffee; DC, dark coffee; DGC, dried green coffee; DRC, dried red coffee; FRC, fresh red coffee; AL, Arabica leaf; RL, Robusta leaf; CP, coffee pulp; MHB, Mueller Hinton Broth. Values are means of triplicate determination (n = 3) ± standard deviations. Significant differences are as follows:*

* *p < 0.05*,

** *p < 0.01*,

*** *p < 0.001 compared with negative control (MHB)*.

We then investigated the MIC and MBC of the extracts on the representatives of 20 *V. cholerae* clinical strains that maintain an antibiotic resistant pattern, such as streptomycin (STM), colistin (COL), nalidixic acid (NAL), sulfamethoxazole (SMX), tetracycline (TET), trimethoprim (TMP), ciprofloxacin (CIP), and azithromycin (AZI). The results, which were expressed as MIC and MBC values in Table 3, show that all of the extracts were active against all of the strains. The most effective against the representative clinical strains had a MIC of 6.25 mg/mL, referring to DGC (65%), DRC (60%), and CP (55%), respectively.

**Table 3:** The susceptibility of a total of 20 *V. cholerae* clinical strains to the extracts of coffee beans and coffee by-products.

**Coffee beans or coffee by-products**	**No. of strains (%)**					
	**MIC [mg/mL]**			**MBC [mg/mL]**		
	**6.25**	**12.5**	**25**	**12.5**	**25**	**50**
LC	2 (10.0)	16 (80.0)	2 (10.0)	0	2 (10.0)	18 (90.0)
MC	4 (20.0)	15 (75.0)	1 (5.0)	0	6 (30.0)	14 (70.0)
DC	1 (5.0)	19 (95.0)	0	0	0	20 (100.0)
DGC	13 (65.0)	4 (20.0)	3 (15.0)	0	14 (70.0)	6 (30.0)
DRC	12 (60.0)	7 (35.0)	1 (5.0)	4 (20.0)	9 (45.0)	6 (30.0)
FRC	1 (5.0)	8 (40.0)	11 (55.0)	5 (25.0)	5 (25.0)	10 (50.0)
AL	3 (15.0)	15 (75.0)	2 (10.0)	0	17 (85.0)	3 (15.0)
RL	6 (30.0)	14 (70.0)	0	0	0	20 (100.0)
CP	11 (55.0)	8 (40.0)	1 (5.0)	0	18 (90.0)	2 (10.0)

*LC, light coffee; MC, medium coffee; DC, dark coffee; DGC, dried green coffee; DRC, dried red coffee; FRC, fresh red coffee; AL, Arabica leaf; RL, Robusta leaf; CP, coffee pulp. The bold values indicates MIC and MBC*.

As part of roasted coffee extracts, the trend of MIC and MBC values were determined to be 12.5 (mean 83.33 ± 10.41%) and 50.0 (mean 86.67 ± 15.28%) mg/mL, respectively. The findings of this research show that 95% of the tested strains were sensitive to DC, followed by LC (80%), and MC (75%), respectively. Furthermore, treating P36 *V. cholerae* El Tor Ogawa, which carried five drug resistances, i.e., COL, NAL, SMX, TET, and TMP, with DC gave a MIC value of 6.25 mg/mL (Supplementary Table 2). Similarly, the results also revealed that two of three coffee fruit extracts (DGC and DRC) efficiently suppressed the growth of pathogens, with the MIC at 6.25 mg/mL (65% for DGC and 60% for DRC), while 55% of treated strains expressed a MIC of 25.0 mg/mL after treatment with FRC. Treatment of P46 *V. cholerae* El Tor Ogawa with FRC gave the lowest MIC value at 3.125 mg/mL, despite carrying five drug resistance genes, namely AZI, NAL, SMX, TET, and TMP. Furthermore, Arabica and Robusta coffee leaf extracts with MICs of 12.5 mg/mL suppressed *V. cholerae* growth conditions by 75% (AL) and 70% (RL), respectively, whereas the MIC of CP extract was 6.25 (55%), 12.5 (40%), and 25 (5%) mg/mL. These results suggest that the effects of coffee beans and coffee by-product extracts on antimicrobial activities are varied depending on the extract and the *V. cholerae* clinical strain.

Regarding MDR *V. cholerae* O1, which is a major public health concern, we investigated the synergistic effect of each extract and tetracycline in the N16961 and P48 *V. cholerae* El Tor Ogawa strains, a reference and six-drug resistance strain (AZI, COL, NAL, SMX, TET, and TMP), respectively. In double-dose response (checkerboard) experiments, the extract combinations were used to determine the nature of their interaction with tetracycline. Table 4 shows the evaluation of the synergistic effect of the extracts and antibiotics. In both standard and multidrug resistance strains, the combination effect on bacterial growth appeared to be greater than treatment alone, with FICI ranging from 0.005 to 0.258. It is important to note that CP had the lowest FIC index (0.005) when combined with 47.5 μg/mL and tetracycline 60 ng/mL. These results indicate that combining the extracts with tetracycline might be a more effective treatment for *V. cholerae* infection.

**Table 4:** Synergistic effect of coffee beans and coffee by-products in combination with tetracycline against standard and multidrug-resistant *V. cholerae*.

**Samples**	**MIC (mg/mL) of extracts [a]**		**FIC a**	**MIC (mg/mL) of tetracycline [b]**		**FIC b**	**FICI**	**Outcome**
	**Alone**	**Combination**		**Alone**	**Combination**			
**N16961** ***V. cholerae*** **El Tor O1**								
LC	12.50	0.095	0.0076	0.00039	0.000012	0.031	0.038	Synergistic
MC	6.25	0.046	0.0073	0.00039	0.000048	0.123	0.130	Synergistic
DC	12.50	0.095	0.008	0.00039	0.000006	0.015	0.023	Synergistic
DGC	6.25	0.048	0.0076	0.00039	0.0000975	0.250	0.258	Synergistic
DRC	12.50	0.095	0.0076	0.00039	0.00000038	0.001	0.009	Synergistic
FRC	12.50	0.095	0.0076	0.00039	0.000048	0.123	0.131	Synergistic
AL	25.00	0.190	0.0076	0.00039	0.00000038	0.001	0.009	Synergistic
RL	12.50	0.095	0.0076	0.00039	0.00000038	0.001	0.009	Synergistic
CP	12.50	3.125	0.25	0.00039	0.00000038	0.001	0.251	Synergistic
**P48** ***V. cholerae*** **El Tor O1 Ogawa**								
LC	12.50	0.090	0.0072	0.0625	0.0078	0.125	0.132	Synergistic
MC	6.25	0.090	0.0144	0.0625	0.0039	0.062	0.077	Synergistic
DC	12.50	0.090	0.0072	0.0625	0.000006	0.008	0.015	Synergistic
DGC	6.25	0.0475	0.0076	0.0625	0.0019	0.030	0.038	Synergistic
DRC	12.50	0.0475	0.0038	0.0625	0.0039	0.062	0.066	Synergistic
FRC	12.50	0.090	0.0072	0.0625	0.00012	0.002	0.009	Synergistic
AL	25.00	0.090	0.0036	0.0625	0.00000038	0.016	0.019	Synergistic
RL	12.50	0.090	0.0072	0.0625	0.00000038	0.004	0.011	Synergistic
CP	12.50	0.0475	0.0038	0.0625	0.00006	0.001	0.005	Synergistic

*LC, light coffee; MC, medium coffee; DC, dark coffee; DGC, dried green coffee; DRC, dried red coffee; FRC, fresh red coffee; AL, Arabica leaf; RL, Robusta leaf; CP, coffee pulp; FIC, fractional inhibitory concentration; FICI, fractional inhibitory concentration index*.

### Analysis of Bacterial Killing Kinetics

Considering the main bacteriostatic activity of coffee bioactive compounds, the standard CGA, CA, and caffeine, were applied to the N16961 reference strain. Table 5 shows the MIC and MBC data. The MIC for CGA was 0.5 mg/mL, while CA and caffeine had MICs of 1 mg/mL. In contrast, the MBC of all compounds was >4 mg/mL.

**Table 5:** The MIC and MBC values of coffee phytochemicals of the *V. cholerae* O1 El Tor N16961.

**Phytochemical**	**MIC (mg/mL)**	**MBC (mg/mL)**
CGA	0.5	>4
CA	1	>4
Caffeine	1	>4

*CGA, chlorogenic acid; CA, caffeic acid*.

**Figure 1:**
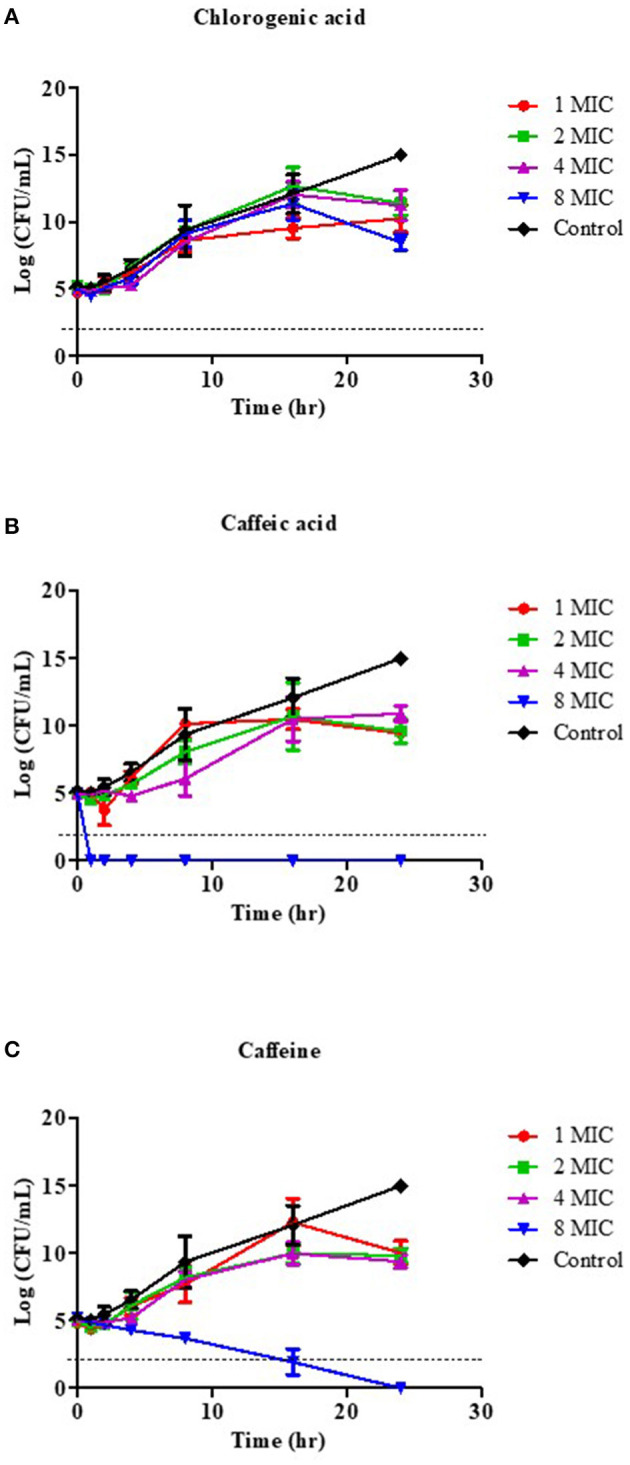
Effect of coffee phytochemical on the viability of *V. cholerae* O1 El Tor N16961. Time-kill kinetics of CGA **(A)**, CA **(B)**, and caffeine **(C)** at concentrations of 1–8 MIC against *V. cholerae* were investigated over a 24 h incubation period at 37°C. The MIC for CGA was 0.5 mg/mL, while the MICs for CA and caffeine were 1 mg/mL. MHB was used as the control instead of compound. Samples were taken at 1, 2, 4, 8, 16, and 24 h to determine viable bacterial numbers. The bactericidal level is indicated by the dashed lines.

We then investigated the time kill kinetics of each coffee phytochemical on the viability of *V. cholerae*, in order to define the bactericidal level using a 1- to 8-fold MIC treatment. Figure 1 demonstrates the time-killing curve analysis. The kill kinetic profiles of the bacterial cultures had no effect when treated with CGA at a concentration of 8x MIC (4 mg/mL) (Figure 1A) or 16x MIC (8 mg/mL), data not shown. Whereas, CA demonstrated rapid bactericidal activity, with an approximate reduction of CFU by 3 log units in viable cell count relative to the initial inoculum at all tested concentrations within 1 h (Figure 1B), 8x MIC (8 mg/mL) of caffeine demonstrated a dose-dependent killing property after 16 h (Figure 1C). As a result, CA may be the most potent bioactive compound in coffee against *V. cholerae*.

### CA Disrupts *V. cholerae* Membrane Permeability

**Figure 2:**
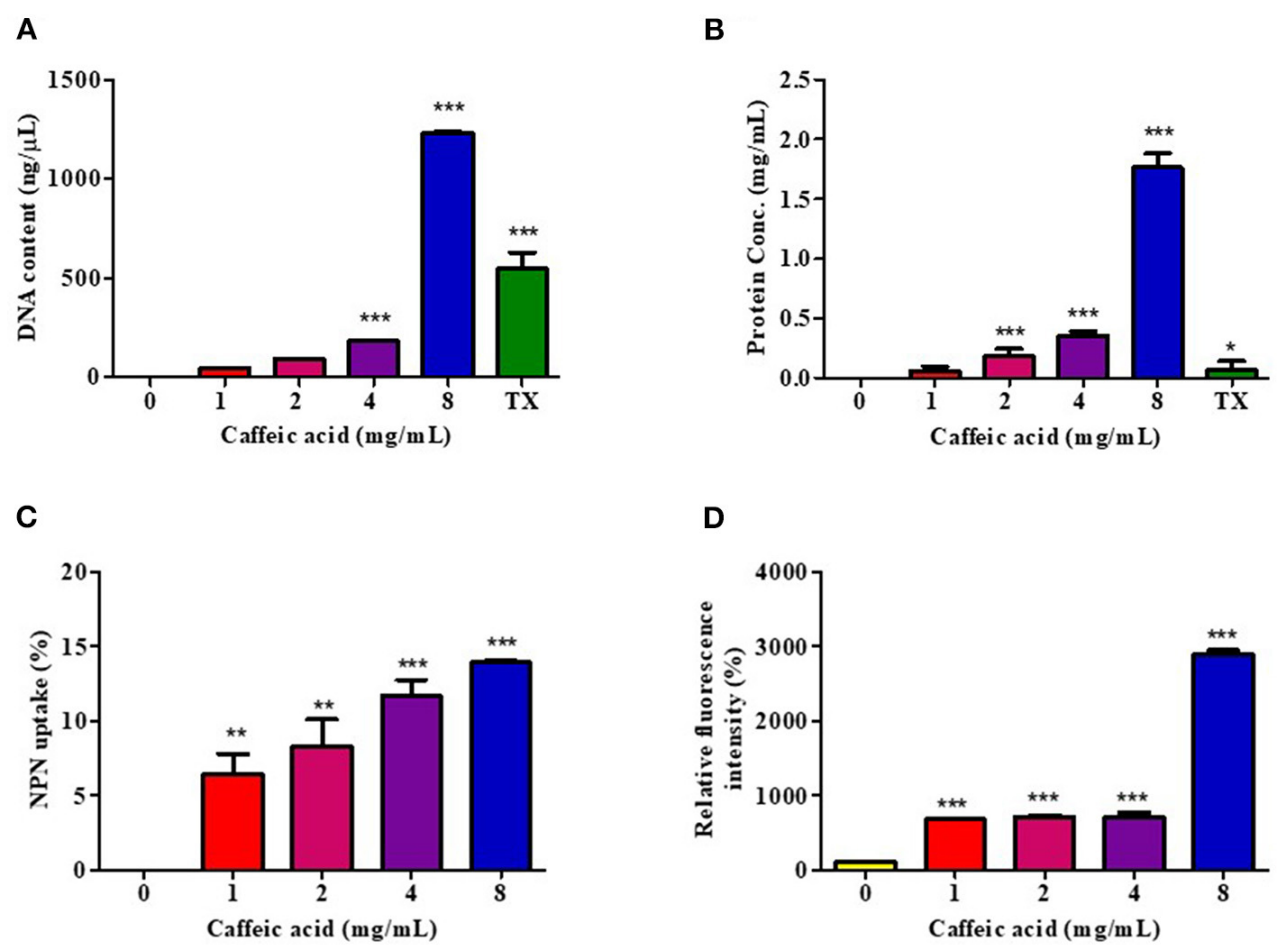
Effect of CA on membrane permeability. *V. cholerae* O1 El Tor N16961 was treated with CA at a concentration of 1–8 mg/mL for 1 h at 37°C. The intracellular leakage of nucleotides **(A)** and proteins **(B)** were measured, and 0.1% Triton X-100 (TX) was used as a positive control. The outer membrane disruption and membrane potential dissipation were investigated by the percentages of NPN uptake **(C)** and Rh123 relative fluorescence intensity **(D)**, respectively. Significant differences compared to untreated controls are indicated by asterisks (*p < 0.05, **p < 0.01, and ***p < 0.001).

To investigate the mechanism of CA on the damaged bacterial cell membrane, an effective drug permeability barrier of the gram-negative cell wall, we measured nucleotide and protein leakage, NPN uptake, and Rh123 incorporation, as shown in Figure 2. Bacterial cells were treated for 1 h with CA at concentrations of 1, 2, 4, and 8 mg/mL, referred to as 1x to 8x MIC.

The leakage of genetic materials, i.e., DNA, and the amount of proteins passing over the cell membrane was used to investigate the effect of CA on the integrity of the membrane via assessment of the absorbance in the CA-treated supernatant. The results are summarized as the DNA content and protein concentration, in Figures 2A,B, respectively, and indicate that the release of cell constituents increased significantly in a CA concentration-dependent manner. Indeed, 8 mg/mL of CA increased DNA and protein leakage more than 0.1% Triton X-100, by about 2.3- and 14.2-fold, respectively.

The NPN uptake assay was used to assess *V. cholerae* outer membrane permeabilization. NPN cannot normally insert into intact bacterial membranes (68); however, when CA disrupts the outer membrane, NPN penetrates the lipid layers, increasing the intensity of its fluorescence emission. CA easily permeabilized the outer membrane in a dose-dependent manner, as indicated by a rise in the intensity of NPN fluorescence (Figure 2C).

We also investigated the transmembrane potential activity by staining Rh123. Considering that Rh123 uptake was proportional to the membrane potential, the results showed that CA treatment increased membrane potential at all tested concentrations (Figure 2D). The highest fluorescence intensity of Rh123 was at 8 mg/mL CA concentration.

These findings suggest that CA may increase membrane potential activity, resulting in increased membrane permeability, which causes intracellular ingredient leakage and cell death.

### CA Altered the Morphological Characterization of *V. cholerae*

**Figure 3:**
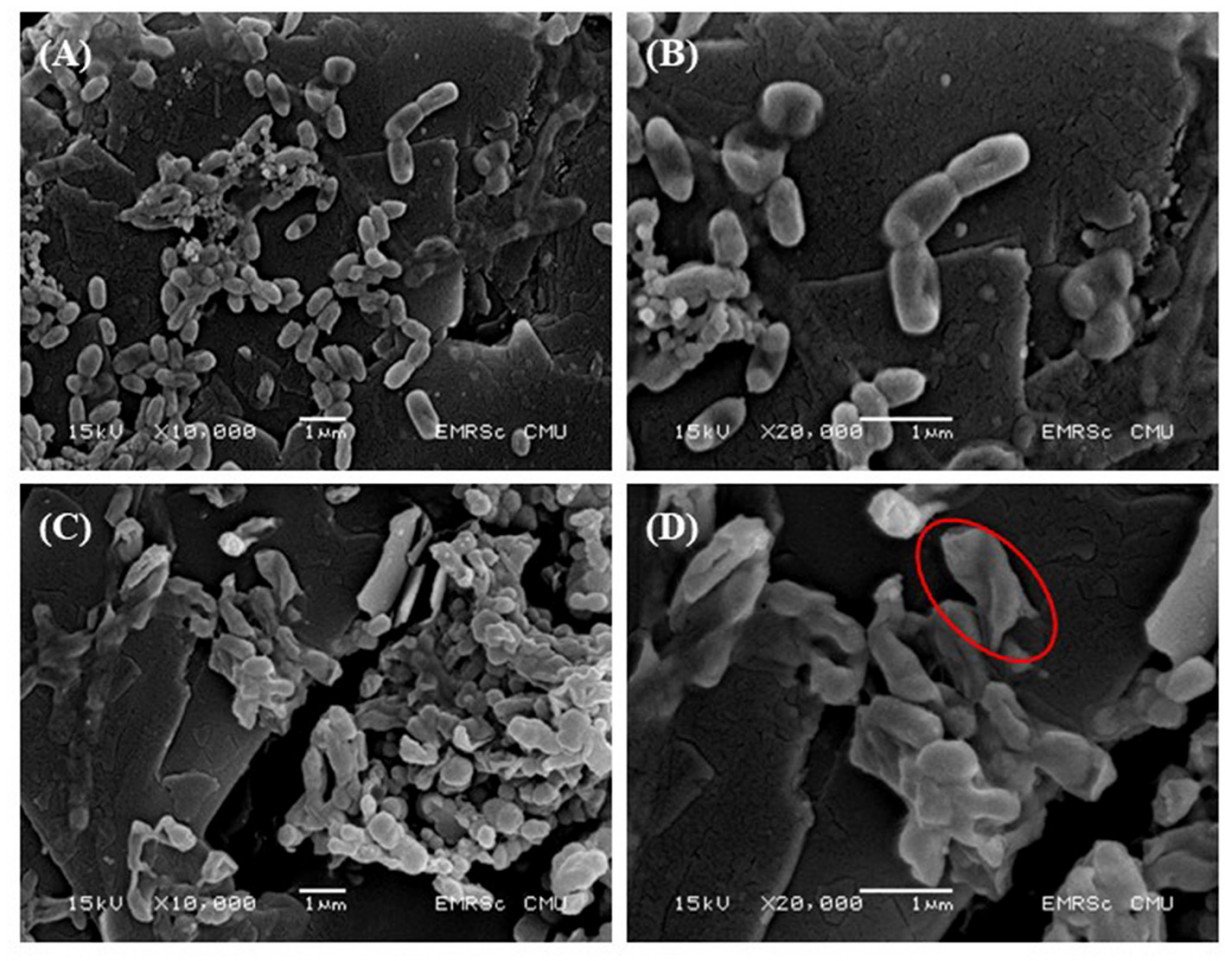
Effect of CA on bacterial cell morphology. *V. cholerae* O1 El Tor N16961 was treated with CA at a concentration of 8 mg/mL for 2 h at 37°C. SEM images at x10,000 and x20,000 magnifications were demonstrated. **(A,B)** Negative controls and **(C,D)** effective treatments. The cell membrane disruption is represented by the red circles.

Finally, SEM was used to compare morphological changes in the appearance of cells with and without 8 mg/mL of CA exposure. Figure 3 shows the SEM images of bacterial cells at x10,000 and 20,000 magnifications. The untreated control bacteria had a smooth, compact surface with an intact cell membrane and no surface ruptures (Figures 3A,B). In contrast, after 2 h of exposure to CA, the cell was found to be severely disrupted (Figure 3C), with membrane corrugations due to withering wrinkling and damage, as indicated by the red circled portions in Figure 3D. Thus, CA treatment of bacterial cells typically interferes with the integrity of the cell membranes, resulting in morphological changes that allow for intracellular material leakage, cell membrane shrinkage, and ultimately cell death.

## Discussion

The efficacy of antibiotics is currently decreasing due to an increase in bacterial antimicrobial resistance. According to a WHO report, antimicrobial resistance is one of the top 10 global public health threats facing humanity due to the misuse and overuse of drugs, including anti-cholera drugs (69). As a consequence, alternative therapeutic approaches are in high demand. Here, we have demonstrated that coffee beans and coffee by-products extract have anti-cholera properties, and CA showed the most effective treatment for *V. cholerae* by involving membrane permeability disruption.

Coffee and its bioactive compounds have been shown to have a variety of pharmacologically beneficial effects on humans. The phytochemical profiles of the extracts tend to vary considerably in terms of CGA and CA content, but not in terms of caffeine. It should be noted that CGA, which has potential anti-MDR *V. cholerae* activity (22–24), was found in the most abundant phenolic compounds in the CP extract, which is consistent with previous findings (70–72). The productivity, chemical composition, and biological activity of coffee extract are all known to be influenced by the brewing process (73). In the current study, three types of roasted coffee beans (light, medium, and dark) were extracted at different temperatures and time durations using ultrasonic-assisted extraction (UAE), an efficient method for retrieving natural antioxidants (30, 48).

Hence, we have demonstrated that roasting circumstances have a considerable impact on the features of the physicochemistry of CGA, but do not significantly affect caffeine thermally, which is consistent with the findings of most of the aforementioned studies (48, 74, 75), while this is the first report of a 2.5-fold decrease in CA in medium and dark coffee. Several studies have previously reported that CGA levels are lost during the roasting process of coffee beans. Using high temperatures during the roasting process has been shown to convert CGA into CGA lactone due to the breaking of carbon-carbon bonds in the CGA structure, resulting in thermal degradation and isomerization (76–78). On the other hand, some studies claim that the level of caffeine increases as the degree of roasting increases, reaching a peak in light and medium-roasted coffee before beginning to decline in dark-roasted coffee. It is anticipated that increasing the temperature can reduce the water content of the coffee beans, thus helping to release volatile compounds (e.g., caffeine) from coffee; indeed, the caffeine levels were reduced significantly compared to the light and medium roast coffee after increasing the temperature to higher limits (dark roast) (79–81).

Our findings are consistent with previous studies, since we demonstrated that green beans (unripe) contain approximately twice as much CGA as red fruits (ripe). The variation in CA content, on the other hand, could be due to a difference in extract solvent: 95% ethanol yielded 38.73 and 26.70 mg/g of green and red fruits, respectively (82). Interestingly, with old coffee leaf, RL provided 6-fold more CGA than AL and 2.3-fold more CA. According to previous studies, Robusta has a higher total phenolic content than Arabica, and old leaf has a higher total phenolic content than young leaf (83–85). Nonetheless, the age of the coffee leaves and the method of processing have an impact on their phytochemical profiles and bioactivity (86).

Although several studies have reported anti-cholera activity with natural product extracts, to the best of our knowledge, this is the first report on the antibacterial activity of coffee beans and coffee-by products extract against *V. cholerae*, particularly with regard to the MDR strain. In this study, we have shown that DGC and DRC fruits, as well as their CP, are the most effective against *V. cholerae*. Furthermore, CP, the first by-product of coffee processing, has been shown to be very effective in the treatment of MDR strains in combination with tetracycline. The effects on antimicrobial activity varied depending on the sample and the clinical strain of *V. cholerae*. However, there is a scarcity of data on the antibacterial activity of coffee beans and coffee by-products extract against *V. cholerae*. Green coffee beans, in particular, had greater antimicrobial activity than roasted coffee. According to many studies, the differences in antibacterial activity between extracts are primarily due to the phenotype and genotype diversity of coffee plants, brewing conditions, roasting temperature, quality of field processing, laboratory extraction processes, and solvents utilized (45, 46, 87). The usefulness of determining the major active components against this bacterium led to a time-killing kinetics study, which revealed that CA had bactericidal activity against *V. cholerae* within 1 h of exposure. This is unexpected, because a previous study found that CGA from *Piper betel* plants had antimicrobial activity against MDR *V. cholerae* at a concentration of the MIC value of 5.5 ± 0.5 mg/mL (22).

CA or 3,4-dihydroxy cinnamic acid, is a phenolic compound found in many plant products, including fruits, wine, coffee, olive oil, and legumes (88). It has been widely used as an alternative strategy to combat microbial pathogenesis and chronic infection caused by microbes such as bacteria, fungi, and viruses, via changing the membrane permeability, inhibition of enzyme activity, damage to the DNA and protein structure, and so on (44). However, the mechanism of antibacterial action of CA in *V. cholerae* has not yet been reported. Many virulent factors are involved in *V. cholerae* infection, including cholera toxin (hemolysins), toxin coregulated plus (TCP), adhesin factor (ACF), hemagglutination-protease (hap, mucinase), neuraminidase, siderophores and outer membrane proteins, and lipopolysaccharides (89). Therefore, the modes of action and target sites of CA might vary considerably. During bacterial infection, the outer membrane prevents the entry of noxious compounds into the cell, helping them recognize the host and facilitate colonization. This prompted us to speculate that CA may influence bacterial membrane permeabilization. As expected, CA disrupted the integrity of *V. cholerae* cell membranes by causing the intracellular material leakage of both proteins and nucleotides, resulting in cell membrane shrinkage and morphological changes that allow for cell death. Similar to a previous study, CA had an effect on the membrane by changing cell permeability, leaking intracellular components, causing membrane damage, and decreasing efflux activity, which has been found in both gram-negative and gram-positive bacteria, such as *Escherichia coli, Pseudomonas aeruginosa*, and *Staphylococcus aureus* (44, 90–92). It is worth noting that the bacterial cells were exposed to a higher concentration of CA (up to 8 mg/mL) than the extract. Because the extract contains CA in concentrations ranging from 0.08 to 2.66 mg/g of extract, the combination effect may be more potent than CA alone. Furthermore, an agar well-diffusion assay used to screen the effect of crude extract revealed that the AL treatment had the largest diameters of inhibition zones, despite the fact that it contained less CA (0.8 mg/g extract). It's possible that this is due to the synergistic effect of CA and other bioactive compounds in the extract. However, this critical point we need to confirm to future study. CA is an excellent synergy compound (93). In drug resistant *Listeria monocytogenes*, 1.5 mg/mL of CA in combination treatment with 50 mg/L of fosfomycin enhanced the antimicrobial activity from 5% of fosfomycin alone to 82% of the fosfomycin and CA combination, which might be by acting as the *FosX* gene inhibitor (94). Besides, CA treatment at 0.5 mg/mL in combination with UV-A LEDs effectively inhibited the survival of foodborne bacteria such as *Escherichia coli* O157: H7, *Salmonella enterica* serovar Typhimurium, and *L. monocytogenes* by inducing cell membrane damage (95).

The cell membrane is an active structure that regulates internal conditions for metabolism and energy transfer. It serves as a primary barrier between the cytoplasm and the extracellular medium. Once this barrier is breached, the bacterial cells cease to function (27). SEM, a powerful tool for investigating the effects of CA on bacterial cells, revealed its inhibitory effects, as confirmed by the severe morphological changes in the tested *V. cholerae*. Similar morphological alterations have also been observed in *V. cholerae* cells treated with the polyphenolic fraction of Kombucha or zinc oxide nanoparticles (96–98). One bacteriostatic mechanism of phenolic compounds is to cause irreversible changes in the cell membrane by altering hydrophobicity and causing local rupture or pore formation in the cell membrane, resulting in an increase in the permeability of the cell membrane, giving rise to the leakage of cellular contents, disrupting the proton-motive force and electron influx, and ultimately destroying cell integrity (99).

Our research has some limitations, because the number of clinical *V. cholerae* strains was small. Furthermore, since CA has synergistic effects with various pharmaceutical entities (93), antimicrobial activity in combination with CGA or caffeine could be tested. This work, however, was intended as a pilot screening, to assess the antibacterial potential of extract against *V. cholerae* clinical drug-resistant strains. For future studies, we need to investigate other modes of action, efficacy, and safety of coffee extracts in animal models and finally in clinical trials.

## Conclusion

The findings of the present study highlight the promising role of the extracts of coffee beans and coffee by-products, especially in combination treatment with tetracycline, as novel anti-cholera compounds, which can be promoted as an alternative therapeutic agent to treat drug-resistant *V. cholerae* infections.

## Data Availability Statement

The original contributions presented in the study are included in the article/Supplementary Material, further inquiries can be directed to the corresponding author.

## Author Contributions

The experiments were conceived and designed by AR, AS, AY, AK, GP-I, OS, SS, and AD. AR, AS, and AD contributed to the experimental design and data analysis. The first draft of the manuscript was written by AR. AR, AS, AY, AK, GP-I, OS, SS, and AD edited the manuscript draft. The published version of the manuscript has been read and approved by all authors.

## Funding

This research was funded by the Unit of Excellence in Research and Product Development of Coffee (Grant No. FF64-UoE64002), and Unit of Excellence on Clinical Outcomes Research and IntegratioN (UNICORN) (Grant No. FF65-UoE005), University of Phayao, Thailand.

## Conflict of Interest

The authors declare that the research was conducted in the absence of any commercial or financial relationships that could be construed as a potential conflict of interest.

## Publisher's Note

All claims expressed in this article are solely those of the authors and do not necessarily represent those of their affiliated organizations, or those of the publisher, the editors and the reviewers. Any product that may be evaluated in this article, or claim that may be made by its manufacturer, is not guaranteed or endorsed by the publisher.
