# Supplementary material for: Potential Antimicrobial Properties of Coffee Beans and Coffee By-Products Against Drug-Resistant Vibrio cholerae
Source: Front Nutr. 2022 Apr 25;9:865684. doi: 10.3389/fnut.2022.865684 (PMC9083461; doi:10.3389/fnut.2022.865684)
Supplement: Supplementary file 1 [file Table_1.DOCX]

**Supplemental data**

**Table S1.** Antimicrobial resistance pattern of *V. cholerae* strains isolated from clinical sample in Thailand.

| **No.** | ***V. cholerae* strains** | **Serogroup** | **Biotypes** | **Year** | **Source** | **Antimicrobial resistance pattern** |
| --- | --- | --- | --- | --- | --- | --- |
| 1 | N16961 (Reference strain) | O1 | El Tor | 1975 | patient | STM |
| 2 | P33 | O1 | El Tor | 2010 | stool | COL, NAL, SMX, TET, TMP |
| 3 | P34 | O1 | El Tor | 2010 | stool | COL, NAL, SMX, TMP |
| 4 | P35 | O1 | El Tor | 2010 | stool | COL, NAL, SMX, TMP |
| 5 | P36 | O1 | El Tor | 2010 | stool | COL, NAL, SMX, TET, TMP |
| 6 | P38 | O1 | El Tor | 2010 | stool | COL, NAL, SMX, TMP |
| 7 | P39 | O1 | El Tor | 2010 | stool | COL, NAL, SMX, TMP |
| 8 | P42 | O1 | El Tor | 2010 | stool | CIP, COL, NAL, SMX, TMP |
| 9 | P43 | O1 | El Tor | 2010 | stool | COL, NAL, SMX, TMP |
| 10 | P44 | O1 | El Tor | 2010 | stool | COL, NAL, SMX, TMP |
| 11 | P46 | O1 | El Tor | 2010 | stool | AZI, NAL, SMX, TET, TMP |
| 12 | P48 | O1 | El Tor | 2010 | stool | AZI, COL, NAL, SMX, TET, TMP |
| 13 | 22115 | O1 | El Tor | 2004 | stool | COL |
| 14 | 22116 | O1 | El Tor | 2004 | stool | COL |
| 15 | 22118 | O1 | El Tor | 2004 | stool | COL |
| 16 | 22125 | O1 | El Tor | 2005 | rectal swab | COL, NAL, SMX, TMP |
| 17 | 22126 | O1 | El Tor | 2005 | rectal swab | COL, NAL, SMX, TMP |
| 18 | 22127 | O1 | El Tor | 2005 | rectal swab | COL, NAL, SMX, TMP |
| 19 | 22136 | O139 | - | 1994 | stool | COL, SMX, TMP |
| 20 | 22137 | O139 | - | 1994 | stool | COL, SMX, TMP |

Abbreviation: AZI, azithromycin; CIP, ciprofloxacin; COL, colistin; NAL, nalidixic acid; SMX, sulfamethoxazole; STM, Streptomycin; TET, tetracycline; TMP, trimethoprim.
