# Supplementary material for: Potential Antimicrobial Properties of Coffee Beans and Coffee By-Products Against Drug-Resistant Vibrio cholerae
Source: Front Nutr. 2022 Apr 25;9:865684. doi: 10.3389/fnut.2022.865684 (PMC9083461; doi:10.3389/fnut.2022.865684)
Supplement: Supplementary file 2 [file Table_2.DOCX]

**Supplemental data**

**Table S2.** The susceptibility of a total of 20 *V. cholerae* clinical strains to coffee beans and coffee by-products

| **Samples** | **MIC** | | | **MBC** |
| --- | --- | --- | --- | --- |
|  | **Conc. (mg/mL)** | **Strains** | **Conc. (mg/mL)** | **Strains** |
| LC | 6.25 | P36, P46 | 12.5 | - |
|  | 12.5 | N16961, P33, P34, P35, P38, P39, P42, P44, P48, 22116, 22118, 22125, 22126, 22127, 22136, 22137 | 25 | P42, 22115 |
|  | 25 | P43, 22115 | 50 | N16961, P33, P34, P35, P36, P38, P39, P43, P44, P46, P48, 22116, 22118, 22125, 22126, 22127, 22136, 22137 |
| MC | 6.25 | N16961, P36, P39, P46 | 12.5 | - |
|  | 12.5 | P33, P34, P35, P38, P42, P43, P44, P48, 22116, 22118, 22125, 22126, 22127, 22136, 22137 | 25 | P34, P35, P42, 22115, 22116, 22118 |
|  | 25 | 22115 | 50 | N16961, P33, P36, P38, P39, P43, P44, P46, P48, 22125, 22126, 22127, 22136, 22137 |
| DC | 6.25 | P36 | 12.5 | - |
|  | 12.5 | N16961, P33, P34, P35, P38, P39, P42, P43, P44, P46, P48, 22115, 22116, 22118, 22125, 22126, 22127, 22136, 22137 | 25 | - |
|  | 25 |  | 50 | N16961, P33, P34, P35, P36, P38, P39, P42, P43, P44, P46, P48, 22115, 22116, 22118, 22125, 22126, 22127, 22136, 22137 |
| CGD | 6.25 | N16961, P33, P35, P38, P39, P42, P44, P46, P48, 22125, 22126, 22127, 22136 | 12.5 | - |
|  | 12.5 | P34, P36, P43, 22137 | 25 | P35, P38, P39, P42, P43, P44, P46, P48, 22115, 22116, 22125, 22126, 22127, 22136 |
|  | 25 | 22115, 22116, 22118 | 50 | N16961, P33, P34, P36, 22118, 22137 |
| CRD | 6.25 | P33, P35, P36, P38, P39, P42, P44, P48, 22125, 22126, 22127, 22136, | 12.5 | P33, 22125, 22126, 22127 |
|  | 12.5 | N16961, P34, P43, P46, 22115, 22118, 22137 | 25 | P34, P35, P36, P38, P39, 22115, 22116, 22118, 22136 |
|  | 25 | 22116 | 50 | N16961, P42, P43, P44, P46, P48 |
| CRF | 3.125 | P46 | 12.5 | P36, P46, 22115, 22116, 22118 |
|  | 6.25 | P34, P35, P36, P38, P39, P42, 22126, 22127 | 25 | N16961, P34, P48, 22126, 22127 |
|  | 12.5 | N16961,P33, P43, P44, P48, 22115, 22116, 22118, 22115, 22136, 22137 | 50 | P33, P35, P38, P39, P42, P43, P44, 22125, 22136, 22137 |
| AL | 6.25 | 22115, 22116, 22118 | 25 | - |
|  | 12.5 | P33, P34, P35, P36, P38, P39, P42, P43, P44, P46, P48, 22125, 22127, 22136, 22137 | 50 | N16961, P34, P35, P38, P39, P42, P43, P44, P46, P48, 22115, 22116, 22118, 22125, 22127, 22136, 22137 |
|  | 25 | N16961, 22126 | 50 | P33, P36, 22126 |
| RL | 6.25 | P34, P36, P39, 22115, 22116, 22118 | 12.5 | - |
|  | 12.5 | N16961, P33, P35, P38, P42, P43, P44, P46, P48, 22125, 22126, 22127, 22136, 22137 | 25 | - |
|  | 25 | - | 50 | N16961, P33, P34, P35, P36, P38, P39, P42, P43, P44, P46, P48, 22115, 22116, 22118, 22125, 22126, 22127, 22136, 22137 |
| CP | 6.25 | P35, P38, P39, P42, P44, P46, P48, 22125, 22127, 22136, 22137 | 12.5 | - |
|  | 12.5 | N16961, P33, P34, P36, P43, 22116, 22118, 22126 | 25 | N16961, P34, P35, P36, P38, P39, P42, P43, P44, P46, P48, 22115, 22116, 22118, 22125, 22127, 22136, 22137 |
|  | 25 | 22115 | 50 | P33, 22126 |
